# Cross‐sectional areas of deep/core veins are smaller at lower core body temperatures

**DOI:** 10.14814/phy2.13839

**Published:** 2018-08-28

**Authors:** Anna Colleen Crouch, Ulrich M. Scheven, Joan M. Greve

**Affiliations:** ^1^ Department of Mechanical Engineering University of Michigan Ann Arbor Michigan; ^2^ Department of Biomedical Engineering University of Michigan Ann Arbor Michigan

**Keywords:** Cardiovascular system, MRI, thermoregulation

## Abstract

The cardiovascular system plays a crucial role in thermoregulation. Deep core veins, due to their large size and role in returning blood to the heart, are an important part of this system. The response of veins to increasing core temperature has not been adequately studied in vivo. Our objective was to noninvasively quantify in C57BL/6 mice the response of artery‐vein pairs to increases in body temperature. Adult male mice were anesthetized and underwent magnetic resonance imaging. Data were acquired from three colocalized vessel pairs (the neck [carotid/jugular], torso [aorta/inferior vena cava (IVC)], periphery [femoral artery/vein]) at core temperatures of 35, 36, 37, and 38°C. Cross‐sectional area increased with increasing temperature for all vessels, excluding the carotid. Average area of the jugular, aorta, femoral artery, and vein linearly increased with temperature (0.10, 0.017, 0.017, and 0.027 mm^2^/°C, respectively; *P* < 0.05). On average, the IVC has the largest venous response for area (18.2%/°C, vs. jugular 9.0 and femoral 10.9%/°C). Increases in core temperature from 35 to 38 °C resulted in an increase in contact length between the aorta/IVC of 29.3% (*P* = 0.007) and between the femoral artery/vein of 28.0% (*P* = 0.03). Previously unidentified increases in the IVC area due to increasing core temperature are biologically important because they may affect conductive and convective heat transfer. Vascular response to temperature varied based on location and vessel type. Leveraging noninvasive methodology to quantify vascular responses to temperature could be combined with bioheat modeling to improve understanding of thermoregulation.

## Introduction

The body maintains temperature homeostasis by the process of thermoregulation. The human body's ability to thermoregulate is an important coping mechanism to withstand various physiological states and environmental exposures (van der Zee 2002; Tucker et al. 2004; Polderman 2004; Centers for Disease Control and Prevention, 2006; Stewart et al. 2011). The cardiovascular (CV) system plays a vital role in thermoregulation because of its influence on heat transfer via forced convection and conduction by changes in blood distribution (conduction and convection), blood velocity (convection), and proximity of vessels/tissues (conduction) (Fiala and Havenith 2015). Changes in these parameters are influenced by cardiac output, vessel size, blood flow velocity, and pressure. Therefore, to fully understand the CV system's role in thermoregulation, the effect of body temperature on each parameter must be quantified.

Increasing core temperature has been observed to result in: either increases or decreases in cerebral blood flow depending on the temperature range (Nybo et al. 2002a; Qian et al. 2014; Bain et al. 2015), increased cardiac output (Crandall 2008; Siddiqui 2011), and decreased total peripheral resistance (Kuhn and Turner 1959; Crandall 2008). Absent from this previous work was empirical data regarding geometric (size and shape) and functional (deformation across cardiac cycle) changes in core vessels, which are necessary for improved parameterization and validation of mathematical and computational models(Wissler 1998; Bhowmik et al. 2013). Previous work in the lab quantified cardiac output and the arterial response (area and cyclic strain) to increases in core temperature from head‐to‐toe (Crouch et al. 2017). However, data regarding changes in the core (alternatively, deep) veins are still missing from this body of work. Literature suggests that with a decrease in temperature, the cross‐sectional area of core veins in the torso will increase (Shepherd and Vanhoutte 1975). It is proposed that this is due to the shunting of blood away from the periphery and subcutaneous vessels to minimize heat exchanged with the environment and to increase venous return flow while decreasing blood velocity to increase the heat exchanged between artery‐vein pairs, via the process of counter‐current heat exchange (Shepherd and Vanhoutte 1975; Flavahan and Vanhoutte 1988; Vanhoutte 1991).

It is important to study both arteries and veins because of their differential influence on convective and conductive processes as well as counter‐current heat exchange. For example: 65–75% of total blood volume is distributed in the venous system (Rothe 1983; Milnor 1990); in the arteries, there is high‐pressure flow, and thus higher blood flow velocity, compared to the low‐pressure/slower flow in the veins; and, differences in wall composition and thickness (e.g., the amount, location, and orientation of elastin, collagen, smooth muscle cells (SMC)) affect the stress–strain relationship and thus how an artery or vein will respond to stimuli, such as temperature, as well as the thermal conductivity of the vessel wall itself (Mattson and Zhang 2017).

Magnetic resonance imaging (MRI) can be used to noninvasively study core vasculature due to high temporal and spatial resolution. Using C57BL/6 mice (Leon 2005) and MRI, we noninvasively quantified changes in artery‐vein pairs in the neck (carotid/jugular), torso (infrarenal aorta/inferior vena cava (IVC)), and periphery (femoral artery/vein) at four target core temperatures. We hypothesized that as core temperature was increased from minimally hypothermic at 35°C to minimally hyperthermic at 38°C vascular response would vary based on location in the body from cranial to caudal (e.g. neck < torso < periphery) as well as depth (e.g., femoral artery > carotid artery and aorta, jugular and femoral veins > IVC). To our knowledge, these data are the first to empirically quantify the spatially and temporally resolved response of artery–vein pairs of core vasculature to changes in core temperature in vivo from head‐to‐toe. This geometric and functional data could be used to couple bioheat modeling and computational fluid dynamics (CFD) to improve understanding of thermoregulation.

## Methods

All experiments were carried out with local Institutional Animal Care and Use Committee approval. Animals were housed in a room with temperature (22 ± 2°C) and humidity (~27%) control and an alternate 12 h light/dark cycle.

Healthy adult male (12‐ to 20‐weeks‐old, ~22–30 human years (Flurkey et al. 2007)) C57BL/6 mice, purchased from Charles River Laboratories, were used in this study. Male mice were chosen in this initial study of artery–vein pairs because this sex showed the smallest response in the aorta in our previous work (male: 0.019 mm^2^/°C vs. female: 0.024 mm^2^/°C, (Crouch et al. 2017)). Mice were anesthetized with 1.25–2% isoflurane in 1 L/min of oxygen (Constantinides et al. 2011). Animals were imaged in the supine position at 7T using a Direct Drive console (Agilent Technologies, Santa Clara, CA) and a 40 mm inner diameter transmit‐receive volume coil (Morris Instruments, Ontario, Canada). The four target core temperatures, ranging from minimally hypothermic (35°C) to minimally hyperthermic (38°C), were controlled within ±0.2°C using forced convection and were selected to avoid pathological changes (Teng and Hornberger 2011; Duhan et al. 2012). The system included a heater blowing warm air through the bore of the magnet and over the animal, a rectal temperature probe, and a custom‐built proportional‐integral‐derivative (PID) controller (Labview, National Instruments, Austin TX). Heart rate and respiration were monitored (SA Instruments, Stony Brook, NY).

**Figure 1:**
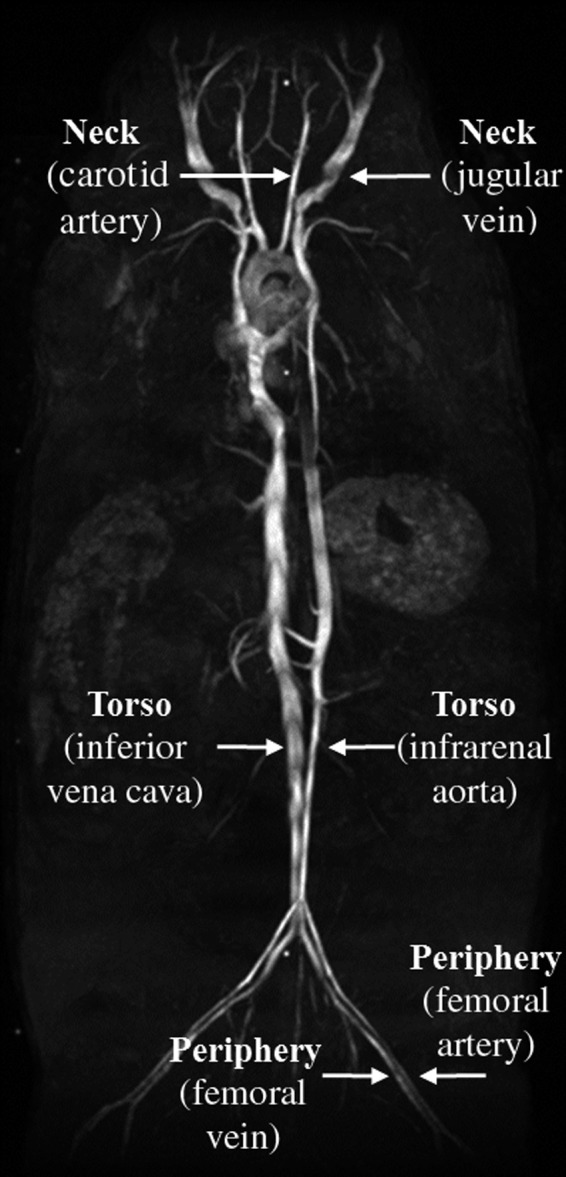
Coronal maximum intensity projection (MIP) (Crouch et al. 2017) illustrating arterial and venous locations where imaging data were acquired and quantified.

Figure 1 illustrates the locations investigated in this study. CINE data were acquired in the neck (carotid artery and jugular vein), torso (infrarenal aorta and inferior vena cava), and periphery (femoral artery and vein). To acquire all locations for a given animal, with four target core temperatures tested at each location, three imaging sessions were required (approximately two hours each). Each region was completed within two weeks before acquiring data from the next region; therefore, ages ranged 1–2 weeks within a region and 2–8 weeks between regions. To verify that changes observed in the vasculature were not due to long exposures to anesthesia, the same data acquisition and analysis procedures were repeated every 30 minutes with animals' core temperature maintained at 37°C for two hours (*n* = 2, adult males). Data were acquired at the carotid artery and jugular vein, aorta and IVC, and femoral artery and vein. To acquire preliminary data regarding the thermal environment while imaging, skin and rectal temperatures were recorded using two tissue implantable thermocouple microprobes, IT‐23 (Physitemp Instruments Inc, Clifton, NJ), inserted subcutaneously on the ventral and dorsal side of mice (*n* = 2, adult males), and a rectal temperature probe. Because the thermocouples are not MRI compatible, a set‐up to mimic the magnet bore was used for testing outside the 5 Gauss line in the MR room. The set‐up included a polyvinyl chloride (PVC) pipe size 6 with approximately same diameter and cut to same length as magnet bore and the same PID controlled heater and fan.

### Neck: carotid artery and jugular vein

Sagittal 2D and axial 3D acquisitions were used to plan slices perpendicular to the carotid artery and to the jugular vein. A cardiac‐gated and velocity compensated 2D CINE sequence with 12 frames was used to acquire data at each location. Parameters for the carotid artery were [TR/TE ~120/4 msec depending on heart rate, flip angle (*α*) 60^o^, FOV (20^ ^mm)^2^, matrix 256^2^ zero‐filled to 512^2^, in‐plane resolution (39 *μ*m)^2^, slice thickness 1 mm, NEX 6]. Parameters for the jugular vein were [TR/TE ~120/6 ms depending on heart rate, *α* 30°, FOV (25.6 mm)^2^, matrix 512 x 256 zero‐filled to 512^2^, in‐plane resolution (50 *μ*m)^2^, slice thickness 1 mm, NEX 6]. The difference in parameters (reduced flip angle and resolution, respectively) reflects the slower blood flow and larger size of the veins. These parameters were optimized in a separate, independent study focused on the venous system and allowed us to compare data from the present work (Palmer et al. 2017). Typically, there were 14 voxels across the carotid artery and 24 voxels across the jugular vein.

The CINE images were analyzed for vessel cross‐sectional area and cyclic strain using an in‐house semiautomated process previously described (Palmer et al. 2017; Crouch et al. 2017). Once the vessel boundary was defined using thresholding, it was verified or adjusted by the user, and area was calculated using polar integration. Green‐Lagrange circumferential cyclic strain was calculated using the following equation with P_dias_ as the in vivo diastolic perimeter and P_i_ as the vessel perimeter at a given time frame:

$$\begin{array}{c} E_{i}=\frac{1}{2}\left[{\left(\frac{P_{i}}{P_{\mathrm{dias}}}\right)}^{2}-1\right]\times 100\%\;\text{with i}\to 1-12\;\;\;\;\;\left(1\right) \end{array}$$

### Torso: infrarenal aorta and inferior vena cava (IVC)

Coronal 2D and sagittal 3D acquisitions were used to plan slices perpendicular to the aorta and to the IVC. Image acquisition parameters and analysis were identical to that performed for the carotid artery and jugular vein. Typically, there were 18 voxels across the artery and 24 voxels across the vein. Using manual tracing along the contiguous border between the artery and vein, the contact length between the aorta and IVC was measured at 35 and 38°C for both systole and diastole from data acquired from the IVC (MRVision, MA).

### Peripheral: femoral artery and vein

Coronal 2D and sagittal 3D acquisitions were used to plan slices perpendicular to the artery and to the vein. Data acquisition and analysis were identical to that performed for the carotid artery and jugular vein. Typically, there were 10 voxels across the artery and 12 voxels across the vein. The contact length between the artery and vein was measured at 35 and 38 °C for systole and diastole from data acquired from the femoral vein (MRVision, MA).

### Statistical analysis

Data are plotted as mean ± standard error (SEM) with individual data points. To compare areas (average, maximum, minimum) or maximum cyclic strain derived from a given vessel, repeated measures one‐way ANOVA was used to test for an overall effect of temperature and Tukey's post hoc test was used to account for multiple comparisons while testing for pairwise differences between temperatures. To investigate the relationship between temperature and area or strain over the full temperature range tested, linear regression was used and fitted slopes were tested for whether they differed from zero. Two methods were used to compare artery‐vein pairs. First, the slopes from the linear regression analysis were compared between colocalized artery‐vein pairs. Second, the percent change per one degree increase in temperature (%/°C) was calculated for average area and maximum cyclic strain of each vessel and compared using two‐way ANOVA with Tukey's post hoc test to determine main group effects of location and vessel type. A relative difference (the percent change) was used to account for differences in the size of anatomical structures. To test if the contact length differed between 35 and 38°C at the aorta/IVC and the femoral artery/vein, a two‐tailed paired *t*‐test was used. Significance was set at *P* < 0.05.

## Results

**Figure 2:**
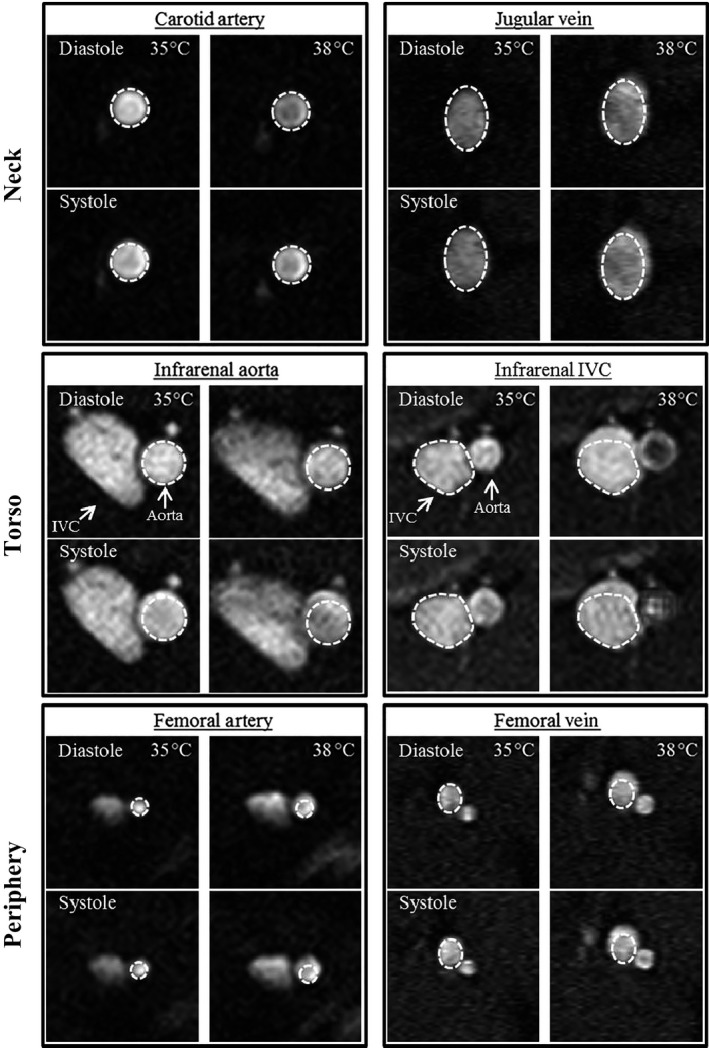
Cross‐sectional view of the artery‐vein pairs at 35 and 38°C with the same‐sized region of interest drawn around the vessel for all four panels (diastole/systole; 35/38°C). IVC: inferior vena cava.

**Figure 3:**
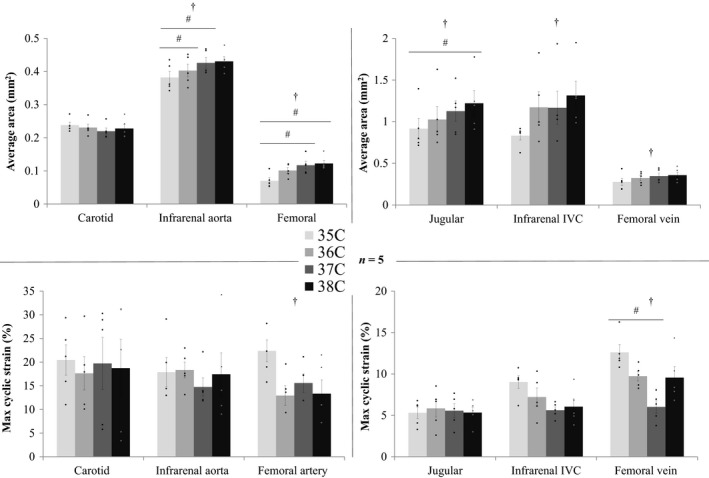
Vessel area averaged (top) and maximum cyclic strain (bottom) across the cardiac cycle for arteries (left) and veins (right) at four core body temperatures: 35, 36, 37, 38 °C (n = 5 adult male mice). Cross‐sectional area increased with increasing temperature for all vessels except the carotid artery. Although not reaching statistical significance in all cases, strain decreases for each vessel from 35 to 38 °C. Significance set at *P* < 0.05: for temperature effect overall (†), pairwise comparison between temperatures for a vessel (#). Individually scaled y‐axes to more clearly illustrate differences among temperature conditions. IVC, inferior vena cava.

**Figure 4:**
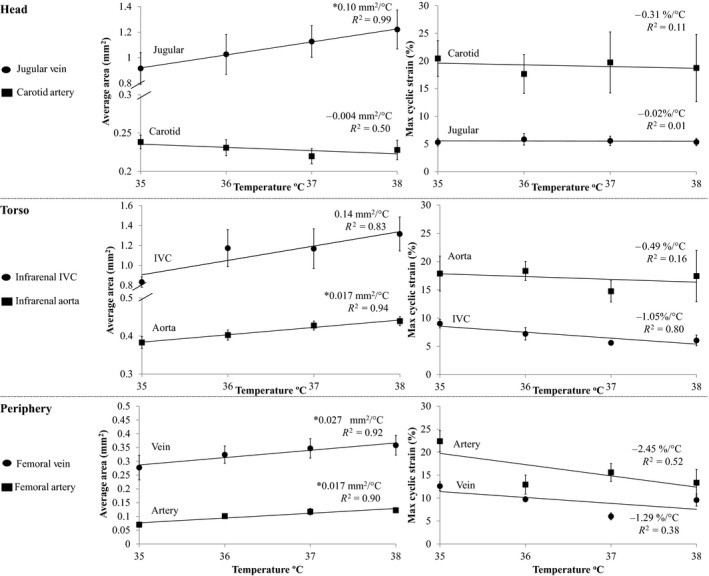
Average vessel area (left) and maximum cyclic strain (right) for artery‐vein pairs at four core body temperatures: 35, 36, 37, 38°C (*n* = 5 adult male mice). Linear regression lines shown for area and strain. Significance set at *P* < 0.05: nonzero slope (*). Individually scaled y‐axes to more clearly illustrate differences among temperature conditions. IVC, Inferior Vena Cava.

Representative images from the three regions of the body, acquired at 35 and 38°C, are shown in Figure 2. Qualitatively, with increasing temperature, there is no discernable increase in either diastolic (minimum) or systolic (maximum) area of the carotid artery. However, there is a noticeable increase in both areas for all other vessels. Figure 3 summarizes area averaged across the cardiac cycle and maximum cyclic strain for the three pairs of colocalized vessels. In Figure 4, average area and maximum cyclic strain are plotted together to highlight their relationship. Table 1 summarizes the results of average area and maximum cyclic strain, including nonsignificant findings. Table S1 summarizes the results of maximum and minimum area response to increasing temperature.

**Table 1:** Summary of results

Location	ANOVA (Fig. 3)		Linear regression (Fig. 4)		
	Overall (*P*‐value)	Pairwise comparisons	Slope (unit/°C)	*R* ^2^	Significance of Slope (*P*‐value)
Average area (mm^2^)					
Carotid	NS (0.07)	None	−0.004 ± 0.003	0.50	NS (0.3)
Jugular	0.006	35/38 *P* = 0.03	0.10 ± 0.003	0.99	0.0007
Aorta	0.02	35/36 *P* = 0.03 35/37 *P* = 0.002	0.017 ± 0.003	0.94	0.03
IVC	0.049	None	0.14 ± 0.05	0.83	NS (0.09)
Femoral Artery	0.002	35/37 *P* = 0.003 35/38 *P* = 0.0004	0.017 ± 0.004	0.90	0.05
Femoral Vein	0.05	None	0.027 ± 0.006	0.92	0.04
Max cyclic strain (%)					
Carotid	NS (0.6)	None	−0.31 ± 0.6	0.11	NS (0.7)
Jugular	NS (0.9)	None	−0.02 ± 0.1	0.01	NS (0.9)
Aorta	NS (0.5)	None	−0.49 ± 0.8	0.16	NS (0.6)
IVC	NS (0.1)	None	−1.05 ± 0.4	0.80	NS (0.1)
Femoral artery	0.03	None	−2.45 ± 1.7	0.52	NS (0.3)
Femoral vein	0.01	35/37 *P* = 0.008	−1.29 ± 1.2	0.38	NS (0.4)

Location comparisons		
Relative changes in average area (%/°C)		
Pairwise (*P*‐value)		
35–36°C	36–37°C (NS)	37–38°C (NS)
IVC>Aorta, 39.2 vs. 5.42%/°C (*P* = 0.04)	Carotid vs. Jugular: −4.75 vs. 12.2%/°C	Carotid vs. Jugular: 3.58 vs. 8.11%/°C
IVC>Carotid, 39.2 vs. −3.23%/°C (*P* = 0.004)	Aorta vs. IVC: 6.00 vs. −0.04%/°C	Aorta vs. IVC: 1.29 vs. 15.5%/°C
Femoral Artery>Carotid, 52.7 vs. −3.23%/°C (*P* < 0.0001)	Femoral Artery vs. Vein: 16.7 vs. 7.63%/°C	Femoral Artery vs. Vein: 6.03 vs. 3.50%/°C
Femoral Artery>Aorta, 52.7 vs. 5.42%/°C (*P* = 0.0009)		
Femoral Artery> Jugular, 52.7 vs. 11.3%/°C (*P* = 0.005)		
Femoral Vein: 21.5%/°C		
Max cyclic strain (%/°C) pairwise (*P*‐value)		
35–36°C (NS)	36–37 °C (NS)	37–38 °C (NS)
Carotid vs. Jugular: −14.9 vs. 23.8%/°C	Carotid vs. Jugular: 4.21 vs. 9.34%/°C	Carotid vs. Jugular: −12.3 vs. −1.03%/°C
Aorta vs. IVC: 8.55 vs. −14.5%/°C	Aorta vs. IVC: −18.6 vs. −17.7%/°C	Aorta vs. IVC: 12.9 vs.
Femoral Artery vs. Vein: ‐40.1 vs. ‐21.2%/°C	Femoral Artery vs. Vein: 26.9 vs. −37.0%/°C	9.85%/°C Femoral Artery vs. Vein: −17.4%/°C vs. 79.7%/°C


Data presented as mean ± SEM, or for location comparisons percent change per degree. Temperature‐interval specific data are listed below with the only significant findings at the 35–36 °C interval, and *P*‐value is denoted in parentheses. IVC: inferior vena cava, NS = not significant.

Heart rate linearly increased with temperature: 515.3 ± 6.8 at 35°C, 533 ± 21 at 36°C, 535.4 ± 24.4 at 37°C, 554.2 ± 24.6 at 38°C (or 11.9 bpm/°C, *P* = 0.03, R^2^=0.93). Two hours of isoflurane exposure at normothermic conditions (37°C) resulted in minimal changes in heart rate (mouse 1: 431, 429, 423, 426 bpm; and, mouse 2: 534, 545, 553, 530 bpm) or vessel area (avg. −0.95%/30 min). With the animal in the supine position, warm air passes over the ventral surface of the animal. Throughout the temperature range tested, ventral and dorsal skin temperature averaged 0.1 and 0.94°C less than core temperature, respectively.

### Cross‐sectional area of the jugular vein increased with increasing core temperature

Temperature did not have an effect on average area of the carotid artery (Fig. 3). Temperature did influence average area of the jugular vein (*P* = 0.006), with statistically significant differences between means at 35/38°C (*P* = 0.03, Fig. 3). The cross‐sectional area of the jugular vein linearly increased with temperature by 0.10 mm^2^/°C, or ~40 voxels/°C (slope > 0, *P* = 0.0007, *R* ^2 ^= 0.99, Fig. 4).

Figure S1 shows cross‐sectional area and cyclic strain across the cardiac cycle for the artery and vein. Cross‐sectional area of the jugular vein, but not the carotid artery, increased with increasing temperature for all timepoints across the cardiac cycle. The average percent change in maximum and minimum areas for the carotid were 2.1 and −0.7%/°C, respectively, and for the jugular were 10.5 and 10.5%/°C, respectively. With minimal change or similar increases in maximum and minimum vessel area in the carotid and jugular, respectively, maximum cyclic strain did not change with core temperature (Fig. 3).

### Cross‐sectional area of the aorta and IVC increased with increasing core temperature

Temperature had an influence on average vessel area for both the aorta (*P* = 0.02, Fig. 3) and IVC (*P* = 0.05, Fig. 3), with statistically significant differences between means for the aorta at 35/36°C and 35/37°C (*P* = 0.03, *P* = 0.002). The average area of the aorta linearly increased by 0.017 mm^2^/°C or ~11 voxels/°C (slope > 0, *P* = 0.03, *R* ^2 ^= 0.94, Fig. 4). Although the slope was not found to be significantly different from zero, the average area of the IVC increased 0.14 mm^2^/°C or ~56 voxels/°C, *R* ^2 ^= 0.83.

Figure S2 shows cross‐sectional area and cyclic strain across the cardiac cycle for the artery and vein. Cross‐sectional area of the aorta and IVC, increased with increasing temperature for nearly all timepoints across the cardiac cycle, with the exception of the IVC at 36 and 37°C where values were nearly identical. The average percent increase in maximum and minimum areas for the aorta were 4.3 and 6.6%/°C, respectively, and for the IVC were 17.9 and 18.8%/°C, respectively. Although the slope was not found to be different from zero, the maximum cyclic strain of the IVC decreased −1.05%/°C, *R* ^2 ^= 0.80.

### Cross‐sectional area of the femoral artery and vein increased with increasing core temperature

Temperature had an influence on average vessel area for the artery (*P* = 0.002) and vein (*P* = 0.05), with statistically significant differences between means for the artery at 35/37°C and 35/38°C (*P* = 0.003 and 0.0004, respectively, Fig. 3). The average area of the femoral artery and vein linearly increased by 0.017 mm^2^/°C or ~11 voxels/°C (slope > 0, *P* = 0.05, *R* ^2 ^= 0.90) and 0.027 mm^2^/°C or ~11 voxels/°C (slope > 0, *P* = 0.04, *R* ^2 ^= 0.92, Fig. 4), respectively. When comparing slopes, the slope of area versus temperature for the femoral vein was larger than for the femoral artery (*P* = 0.02).

Figure S3 shows the cross‐sectional area and cyclic strain across the cardiac cycle for the artery and vein. Cross‐sectional area of the artery and vein increased with increasing temperature for nearly all timepoints across the cardiac cycle. The average percent increase in maximum and minimum areas for the artery were 22.0 and 28.6%/°C, respectively, and for the vein were 10.5 and 11.3%/°C, respectively. Temperature had an influence on maximum cyclic strain for the artery (*P* = 0.03) and the vein (*P* = 0.01), with statistically significant differences between means for the vein at 35/37°C (*P* = 0.008, Fig. 3).

### Response as measured by relative changes in average area was location‐dependent for 35–36°C

Relative changes in average area over a one degree temperature interval (%/°C), compared using two‐way ANOVA with Tukey's post hoc test, were influenced by location and vessel type at the 35–36°C interval (Table 1). The IVC response (39.2%/°C) was 7.4‐fold larger than the aorta response (5.42%/°C, *P* = 0.04) and over 12‐fold larger than the carotid response (−3.23%/°C, *P* = 0.004). The femoral artery response (52.7%/°C) was over 16‐fold larger than the carotid response (−3.23%/°C, *P* < 0.0001), 9.8‐fold larger than aorta response (5.42%/°C, *P* = 0.0009), and 4.6‐fold larger than the jugular response (11.3%/°C, *P* = 0.005). Location and vessel type did not have an effect on response as measured by changes in maximum cyclic strain.

### Contact length between artery and vein increased between 35 and 38°C

**Figure 5:**
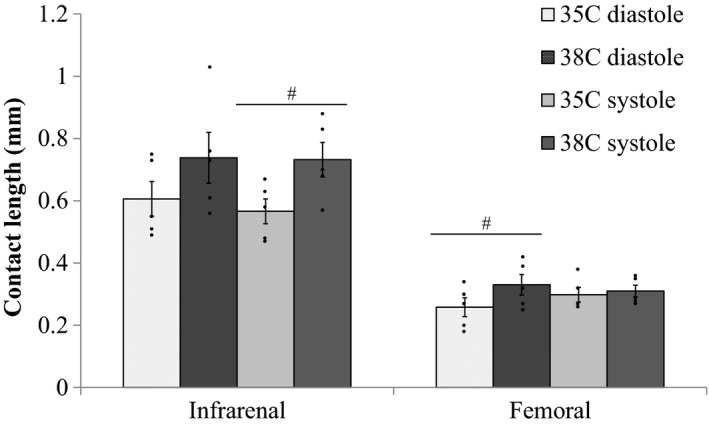
Contact length of contiguous (touching) portion of vessel calculated for core body temperatures of 35 and 38 °C at diastole and systole for the infrarenal and femoral locations (*n* = 5 adult male mice). Significance set at *P* < 0.05 for paired *t*‐test comparison between 35 and 38°C (#).

Qualitatively, the contact length between the infrarenal and femoral artery‐vein pairs increased between 35 and 38°C (Fig. 2). In Figure 5, the contact length is plotted for the aorta/IVC and femoral artery/vein at 35 and 38°C at diastole and systole. Increases in core temperature resulted in an increase of 29.3% in contact length between the aorta and IVC for systole (*P* = 0.007) and an increase of 28.0% in contact length between the femoral artery and vein for diastole (*P* = 0.03).

## Discussion

In contrast to our previous study (Crouch et al. 2017), adult males showed a linear increase in heart rate with increasing temperature. However, the slopes are not statistically different between the two studies (*P* = 0.17), and one slope can be calculated from all 10 animals: 8.3 bpm/°C. Also from our previous study, although not statistically significant, there was a trend of increased stroke volume with increasing temperature (36.1 vs. 37.1 *μ*L/beat, *P* = 0.5 (Crouch et al. 2017)). With an increase in heart rate and either no change or an increase in stroke volume, cardiac output would increase. Temperature‐induced increases in cardiac output would cause an increase in volumetric flow rate in the body, and may also be necessary to maintain blood pressure subsequent to large reductions in total vascular resistance resulting from cutaneous vasodilation (Crandall 2008).

### Femoral artery and infrarenal vena cava exhibit the largest relative changes in area

When comparing the relative vascular response between locations (Table 1), statistically significant differences were seen at the 35–36°C interval. However, different patterns of response between core arteries and veins, based on cranial‐caudal location and depth, are apparent.

Arterial responses to temperature are consistent with previous findings. Lack of change in the carotid artery is reminiscent of our initial work showing minimal vascular response in the Circle of Willis with changes in core temperature. Furthermore, increases in cross‐sectional area of the aorta due to increasing core temperature are in close accord with our previously published data (0.017 mm^2^/°C, Fig. 4; compared to 0.019 mm^2^/°C (Crouch et al. 2017)). As expected, the largest effects of temperature were seen in the femoral artery when comparing relative data (%/°C) (Kuhn and Turner 1959; Crandall 2008). Averaged across the temperature range investigated here, the arterial response increased from cranial to caudal and the more superficial femoral artery had a larger response than the deeper aorta, (carotid −1.4%/°C, aorta 4.2%/°C, and femoral 25.2%/°C; Table 1 and Fig. S1–S3). This would be consistent with the periphery and more superficial vessels being a site for heat exchange with the environment (Kuhn and Turner 1959; Crandall 2008).

Two patterns of response were seen in the veins with respect to cranial‐caudal location. First, relative increases in area were larger in the torso for 35–36°C (jugular, IVC, femoral vein: 11.3, 39.2, 21.5%) and 37–38°C (jugular, IVC, femoral vein: 8.1, 15.5, 3.5%; Table 1). Relative increases in area were larger away from the torso for 36–37°C (jugular, IVC, femoral vein: 12.2, −0.04, 7.6%). An increase in area of the jugular vein with an increase in temperature was expected since there is limited conduction through the skull, and therefore, heat must be removed from the brain via the venous blood (Mariak et al. 1999; Nybo et al. 2002b). Increases in the area of the infrarenal IVC have not been identified previously and would be predicted to affect conductive and convective heat transfer. Changes in the femoral vein reinforce the concept that peripheral and more superficial vessels constrict with decreased temperature to minimize heat loss and, conversely, dilate with increased temperature to maximize heat loss (Kuhn and Turner 1959).

Compared to arteries, veins had a different pattern of response based on depth. Previous research suggests that with a decrease in temperature, core veins in the torso vasodilate to keep blood centrally and away from the skin and periphery where it might lose heat to the environment (Shepherd and Vanhoutte 1975, 1979; Flavahan and Vanhoutte 1988). Researchers hypothesized that this mechanism would lead to an increase in venous return flow with a subsequent decrease in blood velocity, due to increased cross‐sectional area of the vein. This, in turn, would promote heat transfer from the artery to the vein (increased counter‐current heat exchange (Vanhoutte 1991)). Our results illustrate the opposite (Fig. 3 and S2). The infrarenal IVC had the largest relative increase in area for veins (18.2%/°C, compared to 9.0 and 10.9%/°C for the jugular and femoral vein, respectively). This suggests that there is a decrease in venous return flow with decreased temperature. This could result from reduced cardiac output (Crouch et al. 2017) and aortic flow at lower temperatures (as implied by reduced aortic area at lower temperatures). Additionally, recent research has revealed that the enteric nervous system, coined the “second brain”, controls more than just digestion and has large blood flow demands (Gershon 1998; Carabotti et al. 2015). At lower core temperatures, arterial blood may be redistributed to the gastrointestinal tract in order to maintain its temperature. The gut's venous return occurs at a location superior to the infrarenal IVC, and thus, increases in venous return flow with decreases in core temperature may still occur in the IVC superior to the location investigated in this work.

With no other physiological adjustments, the enlargement of the infrarenal IVC with increasing core temperature that we quantified here would act to minimize increases in blood velocity with increased cardiac output (Crandall 2008; Siddiqui 2011; Crouch et al. 2017), and increase counter‐current heat exchange, potentially leading to deleterious effects as more heat is transferred centrally. However, the hemodynamically inactive blood volume (60–70% of total blood volume (Greenway and Lautt 1986)) may decrease with increased core temperature and cardiac output. This would lead to increases in bulk flow at all depths and, thus, greater heat exchange with the environment.

### Increases in contact length between artery‐vein pairs could increase conductive heat transfer

Vessel composition affects the thermal conductivity of the vessel wall. Heat conduction between tissues depends on their thermal conductivities as well as the area of contact between the two tissues. Changes in contact length between artery‐vein pairs due to changing temperature have not been investigated previously. Assuming that contact length also increases proximally and distally to the location at which imaging data were acquired, the increased contact lengths for the aorta/IVC and femoral artery/vein (Fig. 5) suggest an increase in surface area between these artery‐vein pairs. And, heat transfer via conduction would be hypothesized to increase between the artery and vein. Quantifying arterial/venous blood and tissue temperatures, while altering core temperature, will be essential to verify this.

### Considerations for bioheat modeling

Data presented here can direct where and when temperature‐dependent changes in geometry and strain need to be considered when coupling CFD and bioheat modeling (Shitzer et al. 1997; Bhowmik et al. 2013; Coccarelli et al. 2016). For example, the carotid is an anatomical location where you would not need to incorporate geometric changes due temperature. In contrast, changes in geometry would be pertinent for the aorta, femoral artery, and all the veins studied here. With respect to implementation, we show a nearly linear dependence between vessel area and temperature in the jugular vein, infrarenal aorta, femoral artery, and femoral vein (Fig. 4). In the infrarenal IVC there is not a linear relationship due to the large increase between 35 and 36°C and, again, between 37 and 38°C. However, there is a clear overall increase in vessel area with temperature (Fig. S2). A larger temperature range, and thus more data points, is necessary to appropriately fit the nonlinear data. Changes in contact length should also be considered, due to their potential contribution to heat transfer via conduction. Strain data, even if not dependent on temperature, is important for deformable wall models in CFD (Figueroa et al. 2006) and data provided here illustrate large differences in cyclic strain between arteries and veins, which is consistent with previous work (Palmer et al. 2017).

### Limitations

Using murine models has limitations. For example, mice have sweat glands on their paws and studies often require anesthesia. Anesthesia can reduce the core temperature at which physiological responses occur; however, the magnitude of the vascular response is maintained (Støen and Sessler 1990). Also, although isoflurane has vasodilatory effects which can be step‐size and dose dependent, (Matta et al. 1999; Hartley et al. 2007) our monitoring and control of the animals’ physiology allows us to minimize these effects by using low doses and making small adjustments. With temperature well‐controlled under anesthesia, we have begun to separate metabolic, behavioral, and cardiovascular changes due to temperature. We did not, however, measure muscle and skin temperature nor investigate neural or humoral responses. Future studies are required to determine the influence of each of these factors on changes in core vasculature (Frank et al. 1999). Future studies on the effects of cooling and heating the animals using different methods, in contrast to increasing core temperature with air from 35°C to 38°C, would also be of interest to determine which method has the largest impact on the cardiovascular system.

## Conclusions

Challenges in measuring core vasculature have resulted in a lack of empirical information regarding how it might change with core temperature and its role in thermoregulation. Researchers have begun to study the response of core vessels in the leg (Pearson et al. 2011; Chiesa et al. 2016). In our previous work, we expanded this to the whole body for the arterial system in C57BL/6 mice (Crouch et al. 2017), describing geometric and functional changes in the infrarenal aorta that demonstrate the importance of studying core arteries in response to changes in core temperature. Here, we extend our investigations to the venous system by optimizing our noninvasive MRI data acquisition methods. The veins are an important part of the cardiovascular system's role in thermoregulation since blood returning to the heart must be warmed to core temperature via the activation of brown adipose tissue (BAT), countercurrent heat exchange, or other means of heat generation (Leon 2005; Vosselman et al. 2013; Walløe 2016). Although cutaneous veins are undoubtedly involved in thermoregulation (Shepherd and Vanhoutte 1975; Rothe 1983; Rowell 1983), our data show that changes in the IVC, despite its depth, must also be considered. Our most important finding that the cross‐sectional area of the core veins, particularly the IVC, is significantly smaller at lower temperatures is biologically significant due to the potential impact these changes could have on conductive and convective processes involved in thermoregulation. Based on the changes we have quantified here, there is now motivation to further study the core vasculature's response to temperature, including blood velocity and pressure measurements. Our results further motivate research in decreasing core temperature to determine the effect on core vasculature.

## Conflict of Interest

The authors report no conflicts of interest.

## Supporting information




**Figure S1.** Cross‐sectional area (top) and cyclic strain (bottom) across the cardiac cycle for the carotid artery (left) and jugular vein (right).

**Figure S2.** Cross‐sectional area (top) and cyclic strain (bottom) across the cardiac cycle for the infrarenal aorta (left) and inferior vena cava (right).

**Figure S3.** Cross‐sectional area (top) and cyclic strain (bottom) across the cardiac cycle for the femoral artery (left) and vein (right).

**Table S1.** Summary of results for maximum and minimum area with average percent change (%/°C). IV C: Inferior Vena Cava, NS: non‐significant
