# Supplementary figures and images for: Cross‐sectional areas of deep/core veins are smaller at lower core body temperatures
Source: Physiol Rep. 2018 Aug 28;6(16):e13839. doi: 10.14814/phy2.13839 (PMC6113131; doi:10.14814/phy2.13839)

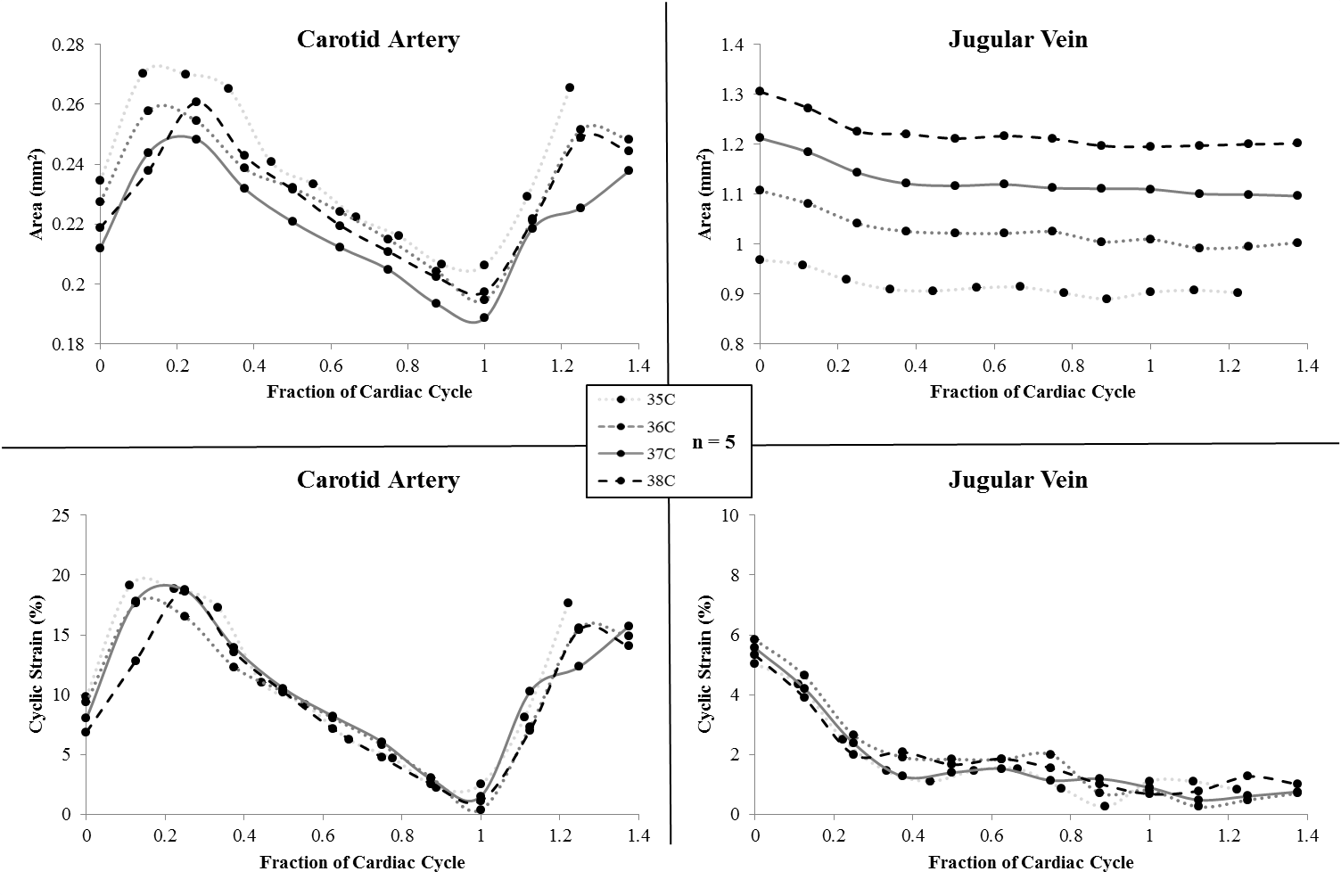

Supplement: Supplementary file 1 — Figure S1. Cross‐sectional area (top) and cyclic strain (bottom) across the cardiac cycle for the carotid artery (left) and jugular vein (right). [file PHY2-6-e13839-s001.png]

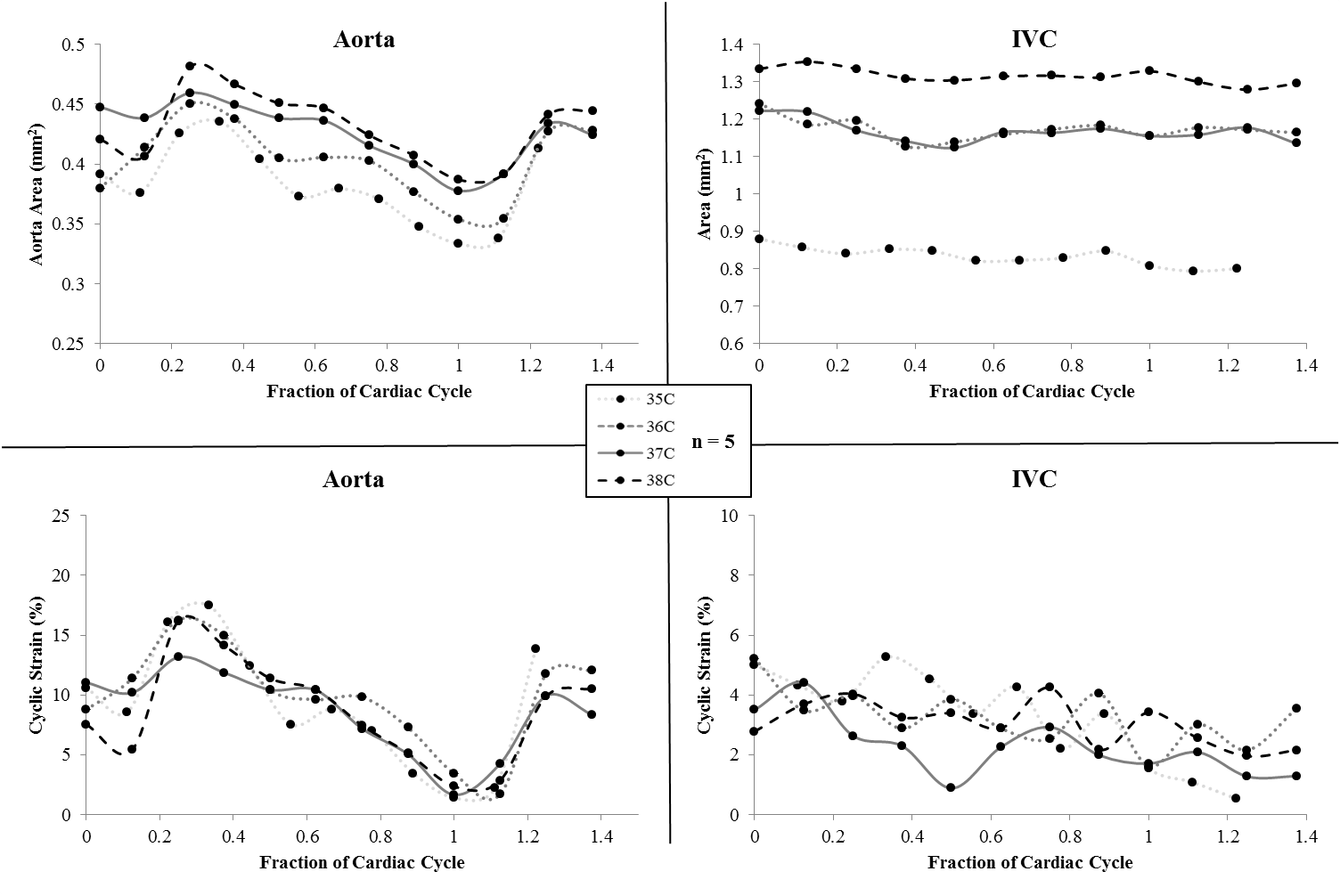

Supplement: Supplementary file 2 — Figure S2. Cross‐sectional area (top) and cyclic strain (bottom) across the cardiac cycle for the infrarenal aorta (left) and inferior vena cava (right). [file PHY2-6-e13839-s002.png]

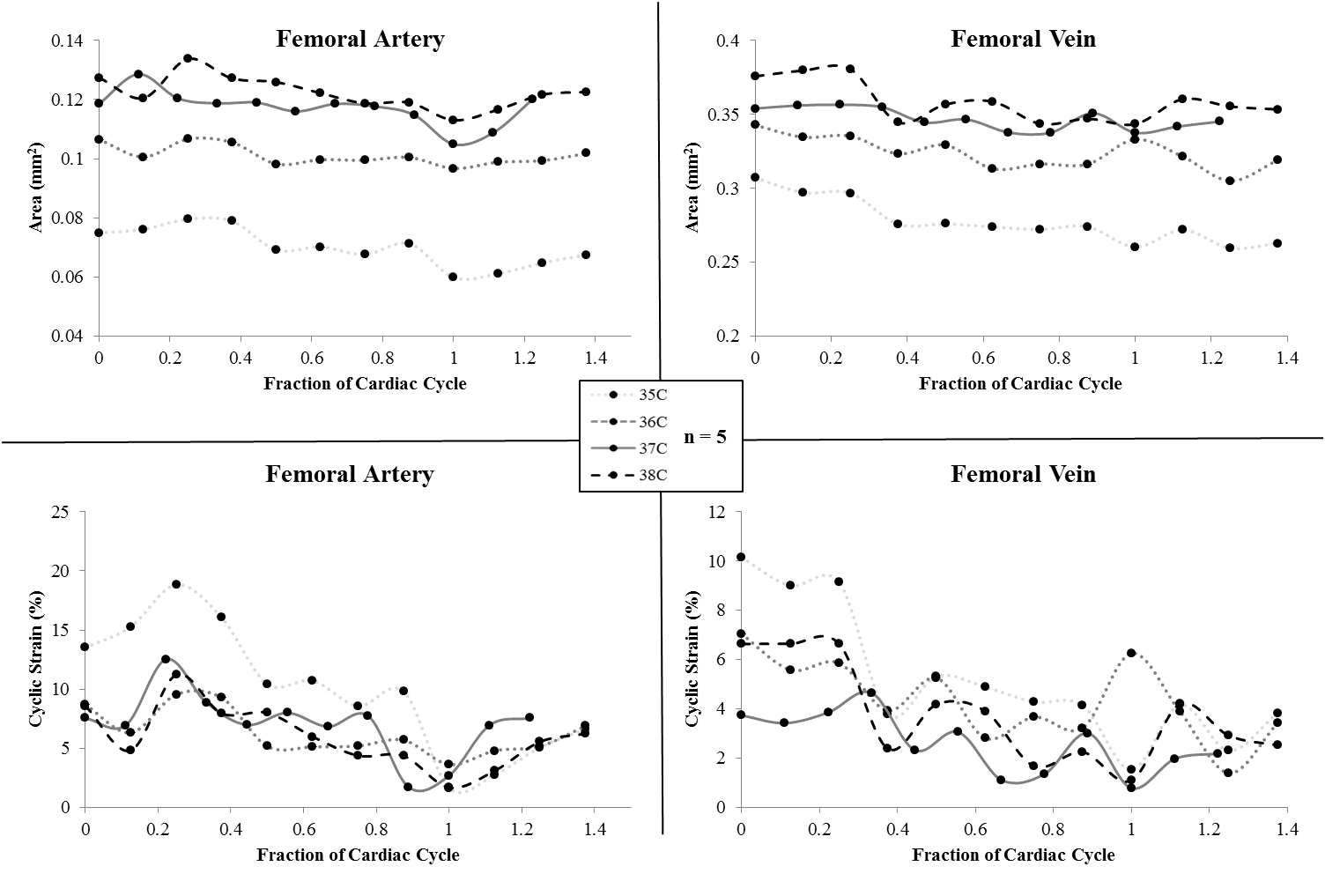

Supplement: Supplementary file 3 — Figure S3. Cross‐sectional area (top) and cyclic strain (bottom) across the cardiac cycle for the femoral artery (left) and vein (right). [file PHY2-6-e13839-s003.png]
