# Supplementary material for: Cross‐sectional areas of deep/core veins are smaller at lower core body temperatures
Source: Physiol Rep. 2018 Aug 28;6(16):e13839. doi: 10.14814/phy2.13839 (PMC6113131; doi:10.14814/phy2.13839)
Supplement: Supplementary file 4 — Table S1. Summary of results for maximum and minimum area with average percent change (%/°C). IV C: Inferior Vena Cava, NS: non‐significant [file PHY2-6-e13839-s004.pdf]

Maximum Area (mm^2)			Minimum Area (mm^2)		
**Location**	**Temperature**		**Location**	**Temperature**	
**Percent Change:** (%/°C)	**Overall** (p-value)	**Pairwise Comparisons**	**Percent Change:** (%/°C)	**Overall** (p-value)	**Pairwise Comparisons**
**Carotid** (-2.1%/°C)	NS (0.19)	None	**Carotid** (-0.7%/°C)	0.03	35/37 p=0.03
**Jugular** (10.5%/°C)	0.006	None	**Jugular** (10.5%/°C)	0.007	35/38 p=0.04
**Aorta** (4.3%/°C)	NS (0.08)	None	**Aorta** (6.6%/°C)	0.009	35/37 p=0.0007<br>35/38 p=0.05
**IVC** (17.9%/°C)	0.04	None	**IVC** (18.5%/°C)	NS (0.06)	None
**Femoral Artery** (22.0%/°C)	0.005	35/37 p=0.01<br>35/38 p=0.002	**Femoral Artery** (28.6%/°C)	0.006	35/37 p=0.0003<br>35/38 p=0.0009
**Femoral Vein** (10.5%/°C)	0.05	None	**Femoral Vein** (11.3%/°C)	0.02	None
