# Supplementary material for: Cross‐sectional areas of deep/core veins are smaller at lower core body temperatures
Source: Physiol Rep. 2018 Aug 28;6(16):e13839. doi: 10.14814/phy2.13839 (PMC6113131; doi:10.14814/phy2.13839)
Supplement: Supplementary file 5 [file PHY2-6-e13839-s005.docx]

Figure S1. Cross-sectional area (top) and cyclic strain (bottom) across the cardiac cycle for the carotid artery (left) and jugular vein (right).

Figure S2. Cross-sectional area (top) and cyclic strain (bottom) across the cardiac cycle for the infrarenal aorta (left) and inferior vena cava (right).

Figure S3. Cross-sectional area (top) and cyclic strain (bottom) across the cardiac cycle for the femoral artery (left) and vein (right).
